# Aortic hemorrhage following anastomotic leakage after esophagogastric surgery before and after implementation of endoscopic vacuum therapy

**DOI:** 10.1007/s00464-025-12029-0

**Published:** 2025-09-10

**Authors:** Lisanne M. D. Pattynama, Roos E. Pouw, Berrie Meijer, Mark I. van Berge Henegouwen, Suzanne S. Gisbertz, Kak Khee Yeung, Jacques J. Bergman, Wietse J. Eshuis

**Affiliations:** 1 Department of Gastroenterology and Hepatology, Amsterdam UMC Location Vrije Universiteit Amsterdam, De Boelelaan 1117, 1081 HV Amsterdam, The Netherlands; 2 Department of Surgery, Amsterdam UMC Location University of Amsterdam, Meibergdreef 9, Amsterdam, The Netherlands; 3 Amsterdam Gastroenterology Endocrinology Metabolism, Amsterdam, The Netherlands; 4 Cancer Treatment and Quality of Life, Cancer Center Amsterdam, Amsterdam, The Netherlands; 5 Dijklander Ziekenhuis, Department of Gastroenterology and Hepatology, Maelsonstraat 3, Hoorn, The Netherlands; 6 Department of Gastroenterology and Hepatology, UMC Location University of Amsterdam, Meibergdreef 9, Amsterdam, The Netherlands; 7 Amsterdam UMC Location Vrije Universiteit Amsterdam and Location, Department of Surgery, University of Amsterdam, Amsterdam Cardiovascular Sciences, Amsterdam, The Netherlands

**Keywords:** Aortic hemorrhage, Esophagectomy, Anastomotic leakage, Endoscopic vacuum therapy

## Abstract

**Background:**

Endoscopic vacuum therapy (EVT) has been established as a safe and effective treatment for anastomotic leakage. While rare, major aortic hemorrhage has been reported as a severe complication potentially associated with EVT. However, significant hemorrhages have also been observed in patients with transmural defects in the upper gastrointestinal tract, without the use of EVT. This raises questions about the role of EVT as a direct cause of aortic hemorrhage.

**Methods:**

The objective of this study was to further investigate the incidence of major aortic hemorrhage in patients with anastomotic leakage following esophagectomy, both before and after the introduction of EVT. This case series included all patients who experienced an aortic hemorrhage after an anastomotic leak over an 11-year period (January 2013–December 2023). Patients were identified from a prospectively maintained database at Amsterdam UMC and were categorized into two groups: the pre-EVT period and the EVT period.

**Results:**

In the pre-EVT period, 355 patients underwent a transthoracic esophagectomy, with 62 (17%) developing anastomotic leakage. In the EVT period, 527 patients underwent the same procedure, with 83 (16%) anastomotic leakages. A total of 7 patients developed a major aortic hemorrhage: 5 in the pre-EVT period (12%) and 2 in the EVT period (2%).

**Conclusion:**

Although EVT has been proposed in literature as a potential cause of major aortic hemorrhage, our findings provide a more nuanced perspective. In this cohort, aortic hemorrhages tend to occur in case of severe mediastinitis, also without treatment with EVT.

**Supplementary Information:**

The online version contains supplementary material available at 10.1007/s00464-025-12029-0.

## Introduction

Esophageal cancer ranks as the seventh leading cause of cancer-related mortality worldwide [1]. Surgical resection remains the cornerstone of treatment for locally advanced esophageal cancer, offering the best chance for cure and long-term survival, despite the risks of significant morbidity, including anastomotic leakage (AL) [2]. Endoscopic vacuum therapy (EVT) is an efficient and safe treatment option for anastomotic leakage following an esophagectomy. For this treatment, an EVT-device with sponge-like material is endoscopically placed over the defect or into an extraluminal cavity. The EVT-device is attached to a vacuum pump, creating negative pressure around the device. Based on negative pressure wound therapy, EVT enhances wound healing, exudate control, and stimulation of perfusion [3]. In recent studies, success rates from 70 to 100% were reported, with complication rates of 10 to 14%, mostly due to stenosis and sponge dislocation [4–6]. Furthermore, Mandarino et al. compared EVT with self-expanding metal stents (SEMS) in patients with upper gastrointestinal anastomotic leaks and demonstrated a higher success rate, shorter treatment duration, less short-term complications, and lower mortality in the EVT-group [7].

Although very rare, major aortic hemorrhage has been described as an adverse event possibly related to EVT [3, 5]. For example, Pournaras et al. reported one patient with a significant bleeding due to a direct aortic branch communication with the cavity during EVT, treated with a covered aortic stent [8]. Furthermore, Ahrens et al. described a patient who deceased due to a major hemorrhage because of an aorto-esophageal fistula at the site of the gastroesophageal anastomosis, 6 weeks after completion of EVT for an anastomotic leakage [9]. Due to the risk of the development of an aortic pseudoaneurysm, leading to major hemorrhage, it has been recommended to avoid EVT in patients with defects close to large vessels [3].

However, the role of EVT as a causative agent in aortic hemorrhage could be questioned, because severe hemorrhage has also been described in patients with a transmural defect in the upper GI tract, without application of EVT [10]. Several studies report rare cases of aortic hemorrhage after foreign body ingestion [11–13]. Furthermore, Matono et al. describe a major aortic hemorrhage in a case of anastomotic leakage after esophagectomy, without the use of EVT [14]. In these cases, infection of the mediastinum is considered as an important factor in development of this severe complication. As patients who undergo EVT generally have a significant mediastinitis, it cannot be ruled out that the infection is a contributing factor in development of a major hemorrhage, rather than the EVT treatment itself.

Timely recognition and imaging of an aorto-esophageal fistula or aortic pseudoaneurysm is crucial for prevention or management of major hemorrhages [13]. However, due to the rare occurrence, management of these complications is not standardized and depends on local clinical experience [13].

In this case series, several cases of a major aortic hemorrhage following anastomotic leakage after esophagogastric surgery in a tertiary referral center are described, before and after implementation of EVT as the preferential treatment for anastomotic leakage.

## Materials and methods

Patients were identified from a prospectively maintained database at the Amsterdam UMC in the Netherlands. Patients with anastomotic leakage after esophagectomy from January 2013 to December 2023 were screened. All patients with an aortic hemorrhage following an anastomotic leak were included in this study. The cases were described according to the available correspondence and medical charts- and were classified into two groups: before and after implementation of endoscopic vacuum therapy. This study was part of a registry study, assessed by the local medical ethics committee, who waived the need for formal ethical review.

### Surgery and EVT procedures

The surgery followed Dutch guidelines for esophagogastric cancer and was performed minimally invasively when possible. The preferred method for esophagectomy was the transthoracic Ivor Lewis procedure, with cervical anastomosis (McKeown procedure) used for proximal tumors or radiation fields, as described in detail elsewhere [15–17]. All surgical patients received a feeding jejunostomy for enteral nutrition.

For patients with anastomotic leakage (AL), standard treatment involved antibiotics, antifungals, proton pump inhibitors, and a nil-by-mouth policy.

In the pre-EVT period, treatment was generally based on the patient's clinical condition and endoscopic evaluation. Small defects in stable patients were treated conservatively with a nasogastric drain. Eligible defects typically received a fully covered metal stent, with or without percutaneous drainage. Larger, contaminated defects in unstable patients required surgery, with a low threshold for surgical intervention.

In the EVT period, all endoscopic vacuum therapy (EVT) procedures were performed under deep propofol sedation or general anesthesia. In the study period, only vacuum-sponges were used for EVT. The use of intracavitary or intraluminal EVT was based on cavity size and extent of debris in the cavity. Sponge exchanges occurred approximately once per week for intraluminal EVT and twice per week for intracavitary EVT. Vacuum pressure ranged from –50 to –125 mmHg, primarily depending on the location of the sponge. The standard EVT procedures have been described in detail previously [18, 19].

## Results

**Fig. 1:**
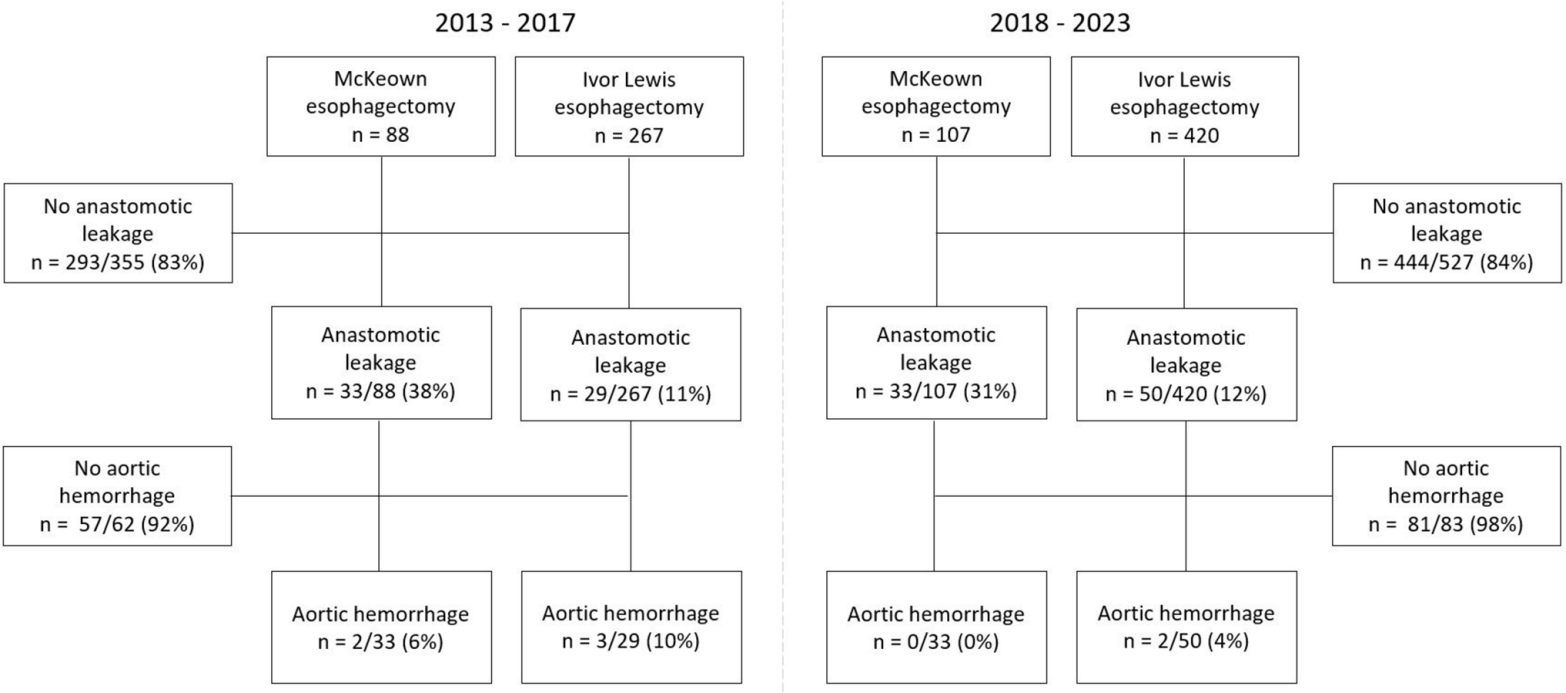
Flowchart of patients who underwent a transthoracic esophagectomy with gastric conduit reconstruction before (2013–2017) and after implementation of EVT (2018–2023)

Between January 2013 and December 2017 (pre-EVT period), 355 patients underwent a transthoracic esophagectomy, of whom 62 (17%) had anastomotic leakage. Between January 2018 and December 2023 (EVT period), 527 patients underwent a transthoracic esophagectomy, with 83 (16%) anastomotic leakages (Fig. 1). Baseline characteristics and treatment of the patients with anastomotic leakage in both periods are displayed in Supplementary Table 1. In the pre-EVT period, 5 AL patients had an aortic hemorrhage, compared to 2 patients in the EVT period (1.4% vs. 0.4%, risk difference = 1.0%, 95% CI =  − 0.3%–2.3%, *p* = 0.097). All 7 cases of aortic hemorrhages are described in this study (Supplementary Table 2).

## Description of cases

### Before implementation of EVT

#### Patient 1

A 65-year-old male underwent neoadjuvant chemoradiotherapy and a McKeown procedure. On POD 3, endoscopy revealed ischemia with necrosis of the tip of the gastric conduit and an anastomotic leak. The patient underwent surgical resection of the ischemic part of the gastric conduit and a redo-anastomosis. After a complicated postoperative period, the patient was discharged to a rehabilitation center on the 71st postoperative day (POD). Four days after discharge, the patient was readmitted after undergoing two consecutive resuscitation interventions in his local hospital, due to hemorrhagic shock. A large aortic pseudo-aneurysm was seen, and it was decided to place an aortic endoprosthesis. During the procedure, the patient deceased. Angiography images revealed contrast in the gastric conduit, presumably due to a fistula between aorta and gastric conduit. No autopsy was performed.

#### Patient 2

**Fig. 2:**
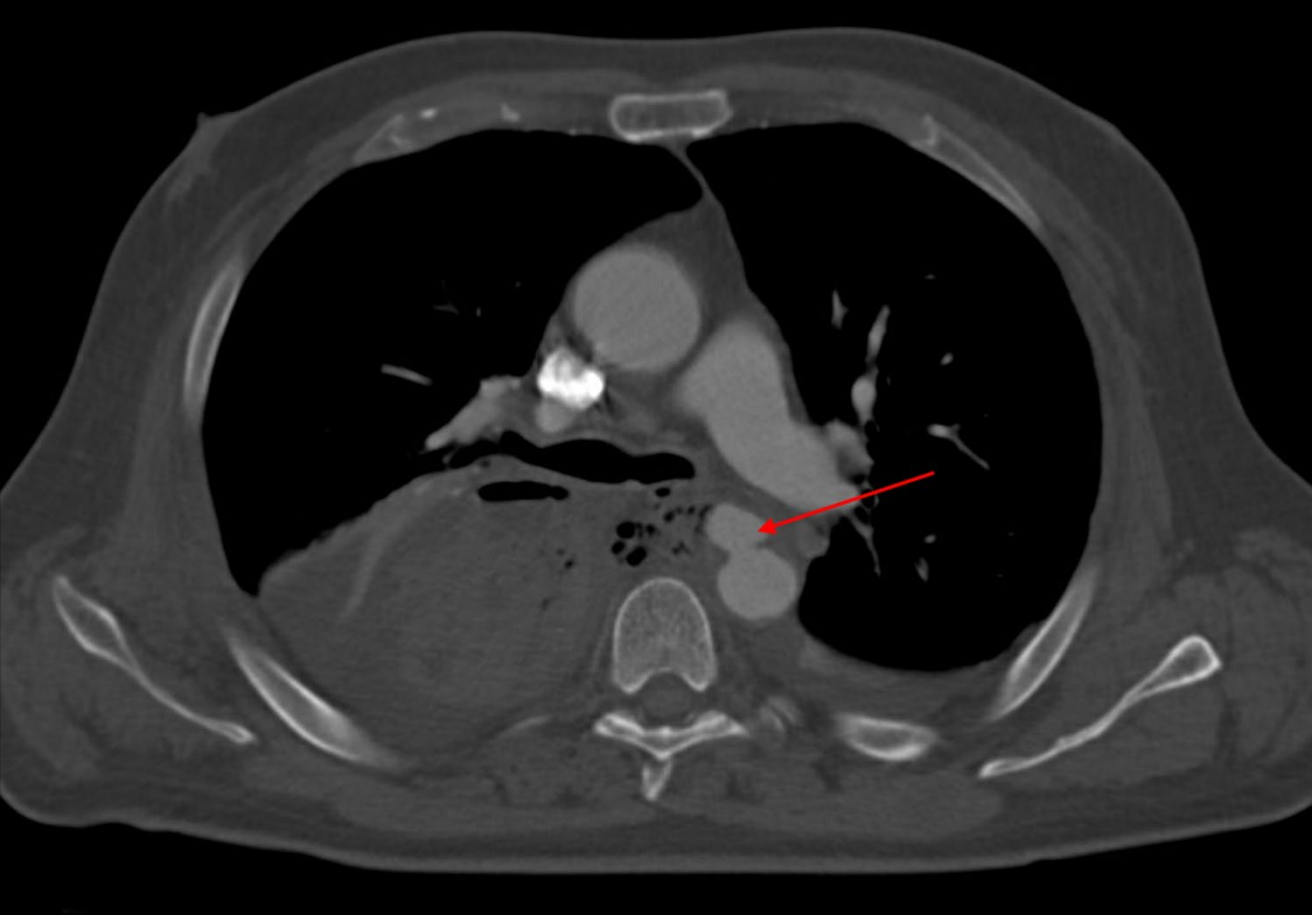
CT angiography showing a pseudoaneurysm in the wall of the thoracic descending aorta adjacent to the gastric conduit (indicated by the red arrow), with adjacent mediastinal air bubbles, indicating mediastinitis

A 56-year-old male underwent neoadjuvant chemoradiotherapy and an Ivor Lewis procedure. After discharge on POD 13, the patient was readmitted with hematemesis on POD 15. CT angiography revealed a pseudoaneurysm of the thoracic descending aorta with active hemorrhage toward the gastric conduit (Fig. 2) and ischemia of the gastric conduit. On the same day, he underwent thoracic endovascular aneurysm repair (TEVAR) and resection of the gastric conduit with reconstruction of a cervical esophagostomy. Additionally, a defect in the left main bronchus was identified intraoperatively, which was covered with a muscle flap. On POD 47, an in-hospital cardiac arrest occurred and resuscitation was successful. Subsequent bronchoscopy revealed a persistent defect in the left main bronchus with a large amount of pus in the mediastinum and lungs. Due to significant neurological impairment after cerebral hypoxia and deteriorating respiratory condition, palliative care was initiated, and the patient passed away on POD 50. No autopsy was performed.

#### Patient 3

A 61-year-old female underwent neoadjuvant chemoradiotherapy and a McKeown procedure. Due to dyspnea, decreasing clinical status and increasing infectious parameters, a CT-scan was conducted, revealing pleural effusion and a mediastinal collection with air configurations, but no leakage of oral contrast. Pleural drainage was performed, but the mediastinal collection could not be reached percutaneously, and CT-scan on POD 12 showed growth of the mediastinal collection. While drainage of the mediastinal collection was being planned, the patient was found unconscious in bed on POD 14. Resuscitation was without success. Autopsy showed a perforation of the longitudinal staple line of the gastric conduit and also of the opposite thoracic aorta, both 3 cm above the diaphragm. Furthermore, approximately, 2 L of blood was encountered in the pleural cavities.

#### Patient 4

A 67-year-old male underwent neoadjuvant chemoradiation and an Ivor Lewis procedure. After discharge on POD 7, he was readmitted on POD 15 with anastomotic leakage and a large mediastinal abscess on CT-scan. Initially, the patient was treated conservatively, including percutaneous drainage of the mediastinal collection. On POD 23, following stagnant clinical improvement, endoscopy showed a near-complete dehiscence of the anastomosis with a vital gastric conduit and a large extraluminal cavity. Due to the size and extent of the defect, it was decided to schedule the patient for reoperation. However, on the morning of POD 25, the patient was found unconscious with 2 L of blood in the drain, and after initial successful resuscitation, the patient developed extensive hematemesis. Subsequent resuscitation was unsuccessful, and the patient deceased due to a presumed aorto-esophageal fistula. No autopsy was performed.

#### Patient 5

A 52-year-old male underwent neoadjuvant chemoradiation and an Ivor Lewis procedure. On POD 13, the patient developed elevated infectious parameters and a mild dyspnea and CT-scan showed an interruption of the anastomotic wall on the medial side, and a mediastinal collection, suspicious of an anastomotic leak. Because of a good clinical status, conservative treatment was initiated, including nasogastric tube drainage. The mediastinal collection was not accessible for percutaneous drainage. In the evening of POD 16, the patient developed acute massive hematemesis and died of hemorrhagic shock despite resuscitation and mass transfusion. The presumptive diagnosis was an aorto-esophageal fistula. No autopsy was performed.

### After implementation of EVT

#### Patient 6

**Fig. 3:**
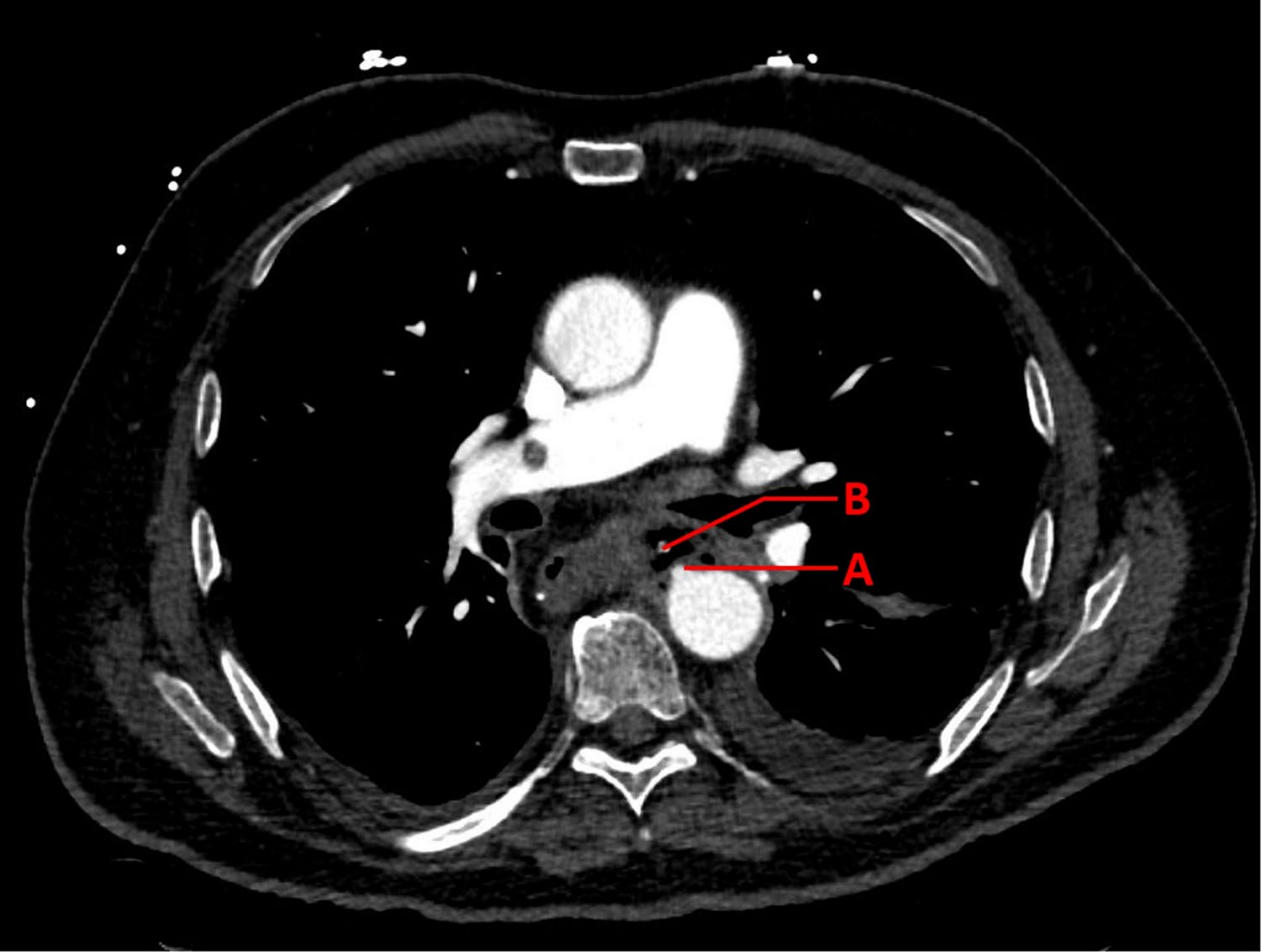
CT angiography with aortic blush (**A**) on the ascending aorta adjacent to the intrathoracic anastomosis with extraluminal vacuum-sponge in the mediastinum (**B**)

A 68-year-old male underwent neo-adjuvant chemoradiotherapy and an Ivor Lewis procedure. Six days post-surgery, the patient developed a sepsis. CT-scan raised suspicion of AL, and subsequent endoscopy revealed ischemia with a partly necrotic proximal gastric conduit. It was decided to initiate EVT with vacuum sponges. After 26 days of EVT and 4 sponge exchanges, the patient had acute hematemesis, and a CT angiography showed a focal irregularity on the ascending aorta close to the extraluminal sponge (Fig. 3). Subsequently, a TEVAR and a re-operation with disconnection of the anastomosis and construction of a cervical esophagostomy were performed. The recovery after the TEVAR involved repeated admissions to the ICU due to multiple complications, including sepsis from the infected aortic stent, atrial fibrillation, and pulmonary embolism. On POD 93, due to the intense treatment and poor prognosis, palliative care was initiated at home by the patient’s request, and he died eight days after discharge.

#### Patient 7

**Fig. 4:**
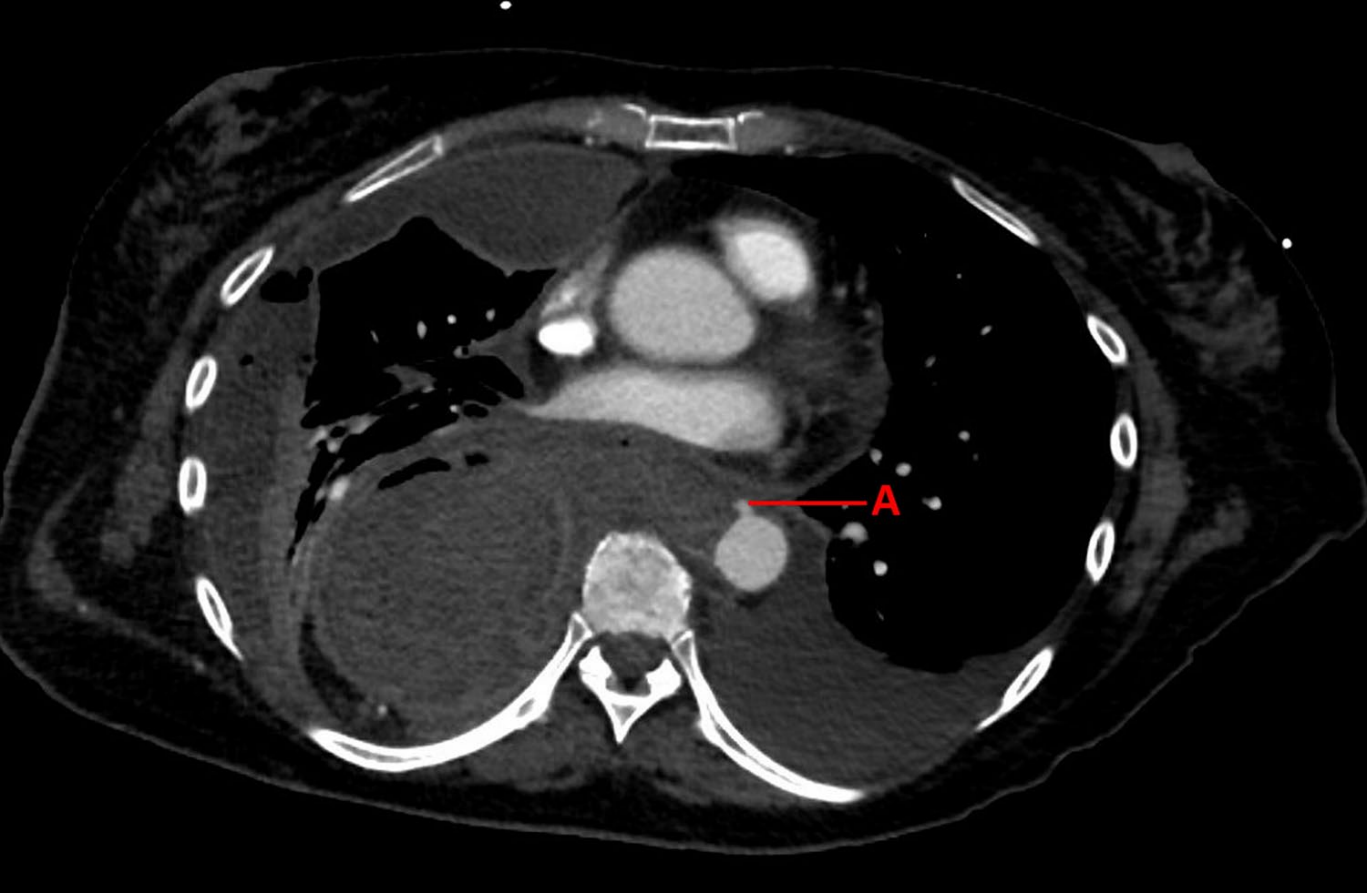
CT angiography with aortic blush (**A**) adjacent to the intrathoracic anastomosis

A 67-year-old female underwent neoadjuvant chemoradiotherapy and an Ivor Lewis procedure. Following discharge on POD 7, the patient was readmitted on POD 10 after collapsing at home. A CT-scan with oral contrast showed anastomotic leakage with extraluminal contrast and an adjacent collection. During endoscopy, dehiscence of 1/3 of the anastomosis was observed and EVT using intracavitary vacuum sponges was initiated, which was switched to intraluminal EVT on POD 21. On POD 26 and after three sponge exchanges in total, the patient had acute hematemesis and melaena. CT angiography showed a blush from the aorta toward the intrathoracic anastomosis (Fig. 4), for which an immediate TEVAR was performed. During the procedure, the patient went into resuscitation setting due to severe hematemesis accompanied by asystole. Moreover, the procedure was complicated by embolisms in the left and right common femoral arteries, treated with embolectomy and Alteplase. Two days after the TEVAR procedure, the patient underwent resection of the gastric conduit with creation of a cervical esophagostomy. In the following weeks, motoric function of the legs was severely impaired, and MRI of the myelum showed spinal cord injury as a complication resulting from the placement of the aortic stent. Given the cause and extent of the injury, the complicated course and the patient’s wish to cease further treatment, it was decided to discontinue further medical intervention. Palliative sedation was initiated, and the patient deceased 46 days after the initial surgery. No autopsy was performed.

## Discussion

The purpose of this study was to list the cases of aorto-esophageal fistulas in patients with AL after esophagectomy, before and after implementation of EVT to evaluate the possible influence of EVT on this severe complication.

As this is a very rare complication, of 882 patients who had underwent a transthoracic esophagectomy during a period of 11 years, only 7 (0.8%) patients were found to suffer from aortic hemorrhage: five before implementation of EVT and two after. The cases are similar to case reports in literature, where major hemorrhage occurred after mediastinitis, with or without EVT treatment [14]. Although aorto-esophageal fistulas have been described as a feared complication of EVT, the specific influence of EVT remains unknown. Multiple cases have previously been reported on the ingestion of a foreign body, causing an esophageal defect with subsequent mediastinitis. In these cases, the ongoing infection led to the development of an aortic pseudoaneurysm, leading to a major hemorrhage in some cases [11–13]. Therefore, aortic hemorrhages appear to result from persistent inflammation in the region rather than from the presence of a continuous vacuum.

In addition to mediastinitis resulting from foreign body ingestion, cases of esophageal foreign bodies causing direct aortic injury have been described in literature. This direct trauma can lead to aortic pseudoaneurysms or aorto-esophageal fistulas [20–22].

Based on this cohort, it seems that aortic hemorrhages tend to occur in severe cases of anastomotic leakage, as some had necrosis of the tip of the conduit, and all patients described here were severely ill and had severe mediastinitis.

Furthermore, in the pre-EVT cases described in this study, 3 out of 5 had significant mediastinal collections that could not be drained percutaneously. With EVT available at that time, these collections might have been effectively drained internally, potentially resulting in a milder presentation of the mediastinitis. Possibly, EVT provides a clean mediastinal environment, which may lead to a more controlled setting.

In cases where a CT angiography was performed, a focal irregularity was seen on the ascending aorta, which can be described as a ‘warning sign.’ When a patient has anastomotic leakage, it can be important to evaluate the aortic area for such signs to possibly prevent or more adequately treat major hemorrhage, whether or not the patient is undergoing EVT treatment. Furthermore, if EVT is initiated and there is any suspicion of close proximity of the EVT-device to large vessels, a CT-scan is recommended to confirm adequate positioning of the EVT-device and timely detect a possible aortic pseudoaneurysm. Additionally, repeat endoscopy should be planned promptly.

This is the first case series that focuses on major hemorrhage from the aorta as a complication of anastomotic leakage after esophagectomy. This study is associated with several limitations. Comparison of cases in two different time periods is associated with bias, as health care has changed significantly. The most important changes are the improvement of surgical techniques, the post-surgical care (i.e., routine controls of inflammatory parameters), advances in imaging, and more efficient collaboration between the departments of Surgery and Gastroenterology. This enables faster and better diagnosis and treatment of anastomotic leakage over the years, making it difficult to isolate the impact of EVT. Furthermore, in clinical practice, autopsy consent is not always granted. Therefore, not all patients underwent an autopsy, limiting the possibility to verify the proposed mechanisms of major aortic hemorrhage.”

This study includes a small number of patients, reducing the statistical power to detect a true difference. Given the rarity of the complication, it is inherently difficult to achieve a larger number of events, which limits the ability to draw more definitive conclusions on this topic.

Although in literature EVT has been described as a possible cause of major aortic hemorrhage, this paper shows a nuance to this hypothesis. This study shows that aortic hemorrhages tend to occur in case of severe mediastinitis, also without treatment with EVT. To avoid the progression to severe mediastinitis, the post operative protocol should focus on fast diagnosis and treatment of severe mediastinitis and gastric conduit necrosis. Due to the severity of the complication, caution is always warranted when using EVT close to the large vessels, and direct contact between the EVT-device and large vessels should be avoided if possible. If there is any doubt about the safety of EVT, a CT-scan should be made after initiation of EVT-to provide more insight.

Further research may help to better understand the relationship between EVT and major aortic hemorrhage. However, given the rarity of this complication, assembling an adequately powered study cohort remains a significant challenge.

## Supplementary Information

Below is the link to the electronic supplementary material.

Supplementary file1 (PDF 105 KB)

Supplementary file2 (PDF 128 KB)
