# Supplementary material for: Aortic hemorrhage following anastomotic leakage after esophagogastric surgery before and after implementation of endoscopic vacuum therapy
Source: Surg Endosc. 2025 Sep 10;39(10):7064–71. doi: 10.1007/s00464-025-12029-0 (PMC12500753; doi:10.1007/s00464-025-12029-0)
Supplement: Supplementary file 1 — Supplementary file1 (PDF 105 KB) [file 464_2025_12029_MOESM1_ESM.pdf]

Supplementary Table 1. Baseline characteristics and treatment of patients with anastomotic leakage

	**Pre-EVT period (n = 62)**	**EVT period (n = 83)**	**P-value**
Age in years, mean (SD)	65 (8)	64 (11)	0.799
Sex ratio (M:F), n (%)	46:16 (74:26)	69:13 (83:17)	0.231
Procedure, n (%)			
Ivor Lewis	29 (47)	50 (60)	0.107
McKeown	33 (53)	33 (40)	
Treatment of AL, n (%)			
Conservative management only	16 (26)	12 (14)	
Stent	11 (18)	1 (1)	
Endoscopic drainage	10 (16)	0 (0)	<0.001
Surgery	25 (40)	11 (13)	
EVT	0 (0)	59 (71)	
