# Supplementary material for: Aortic hemorrhage following anastomotic leakage after esophagogastric surgery before and after implementation of endoscopic vacuum therapy
Source: Surg Endosc. 2025 Sep 10;39(10):7064–71. doi: 10.1007/s00464-025-12029-0 (PMC12500753; doi:10.1007/s00464-025-12029-0)
Supplement: Supplementary file 2 — Supplementary file2 (PDF 128 KB) [file 464_2025_12029_MOESM2_ESM.pdf]

Supplementary Table 2. Baseline characteristics and treatment course per patient

	Age	Gender	Procedure	POD AL diagnosis	Cultures	Treatment AL	POD hemorrhage	Treatment hemorrhage	Course
**Pre-EVT period**									
**1**	65	Male	McKeown	3	Yeasts	Resection ischemic gastric conduit and redo-anastomosis	75	Multiple resuscitations	Deceased on POD 75 during placement of aortic endoprosthesis.
**2**	56	Male	Ivor Lewis	15	Staphylococcus aureus, Streptococcus milleri, Candida albicans	Resection ischemic gastric conduit with cervical esophagostomy	15	TEVAR	Sepsis, cardiac arrest, palliative care due to deterioration of clinical status. Deceased on POD 50.
**3**	61	Female	McKeown	14	Not available	Not applicable	14	Not applicable	Deceased on POD 14.<br>Autopsy showed perforation of the longitudinal staple line of the gastric conduit and also of the opposite thoracic aorta
**4**	67	Male	Ivor Lewis	15	Proteus mirabilis, Streptococcus constellatus, Candida albicans/dubliniensis	Conservative and patient was scheduled for surgery	25	Initially successful resuscitation	Extensive hematemesis: subsequent resuscitation was unsuccessful. Deceased on POD 16.
**5**	52	Male	Ivor Lewis	14	Not available	Conservative (mediastinal collection was not accessible for percutaneous drainage)	16	Not applicable	Acute massive hematemesis, deceased due to hemorrhagic shock on POD 93.
**EVT-period**									
**6**	68	Male	Ivor Lewis	6	Enterococcus faecalis, Enterococcus faecium, Staphylococcus epidermides	EVT and re-operation with disconnection of the anastomosis and construction of a cervical esophagostomy	31	TEVAR	On POD 93, due to the intense treatment and poor prognosis, palliative care was initiated at home by the patient's request. Deceased on POD 101.
**7**	67	Female	Ivor Lewis	10	Enterococcus faecium, oropharyngeal flora	EVT and resection of the gastric conduit with creation of a cervical esophagostomy	26	Successful resuscitation and TEVAR	Due to cause and extent of injury and complications palliative sedation was initiated. Deceased on POD 46.

Abbreviations: POD, post-operative day; AL, anastomotic leakage; EVT, endoscopic vacuum therapy; TEVAR, thoracic endovascular aortic repair.
